# Hybrid Neuromuscular Training Improves Cardiometabolic Health and Alters Redox Status in Inactive Overweight and Obese Women: A Randomized Controlled Trial

**DOI:** 10.3390/antiox10101601

**Published:** 2021-10-12

**Authors:** Alexios Batrakoulis, Athanasios Z. Jamurtas, Dimitrios Draganidis, Kalliopi Georgakouli, Panagiotis Tsimeas, Athanasios Poulios, Niki Syrou, Chariklia K. Deli, Konstantinos Papanikolaou, Symeon Tournis, Ioannis G. Fatouros

**Affiliations:** 1 Department of Physical Education and Sport Sciences, University of Thessaly, 42100 Trikala, Greece; abatrakoulis@uth.gr (A.B.); ajamurt@uth.gr (A.Z.J.); dimidraganidis@gmail.com (D.D.); ptsimeas@pe.uth.gr (P.T.); apoulios@pe.uth.gr (A.P.); nikisyrou@pe.uth.gr (N.S.); delixar@uth.gr (C.K.D.); kpapanikolaou@uth.gr (K.P.); 2 Department of Nutrition and Dietetics, University of Thessaly, 42100 Trikala, Greece; kgeorgakouli@gmail.com; 3 Laboratory for Research of the Musculoskeletal System “Th. Garofalidis”, National and Kapodistrian University of Athens, 14561 Athens, Greece; stournis@med.uoa.gr

**Keywords:** antioxidant capacity, blood lipids, glycemic control, interval exercise training, metabolic syndrome severity

## Abstract

This randomized controlled trial investigated the effects of a 5-month high-intensity hybrid-type neuromuscular training program with nontraditional implements on cardiometabolic health, redox status, and cardiovascular disease (CVD) risk in inactive overweight and obese women. Forty-nine inactive female participants with overweight and obesity (age: 36.4 ± 4.4 years; BMI: 29.1 ± 2.9 kg/m^2^) were randomly assigned to either a control (C, *n* = 21) or a training group (TR, *n* = 28). TR followed a 20-week supervised, progressive, time-efficient (3 days/week; 6–15 min net exercise time) program implementing loaded fundamental movement patterns with prescribed work-to-rest time intervals (20–40 s, 1:2, 1:1, 2:1) in a circuit fashion (2–3 rounds). Cardiometabolic risk factors were measured at baseline and post-training as secondary outcomes of a larger randomized controlled trial. At post-intervention, TR demonstrated favorable changes in resting heart rate (−7%, *p* = 0.043), high-density lipoprotein (+18.1%, *p* = 0.029), atherogenic index (−17%, *p* = 0.045), mean arterial pressure (−4.5%, *p* = 0.03), waist circumference (−6.2%, *p* = 0.005), waist-to-hip ratio (−4.6%; *p* = 0.015), metabolic syndrome severity score (−222%, *p* = 0.024), full 30-year CVD risk (−15.8%, *p* = 0.002) and hard 30-year CVD risk (−17.6%, *p* = 0.01), vascular age (−7.8%, *p* = 0.002), protein carbonyls (−45.7%, *p* = 0.001), catalase activity (+15.2%, *p* = 0.023), and total antioxidant capacity (+11.4%, *p* = 0.002) relative to C. Additionally, TR induced beneficial changes in fasting glucose (−3.4%, *p* = 0.002), homeostatic model assessment for insulin resistance (−15.7%, *p* < 0.001), diastolic blood pressure (−5.6%, *p* < 0.001), reduced glutathione (+39.8%, *p* < 0.001), 10-year CVD risk (−17.4%, *p* = 0.011), and total bilirubin (−21.7%, *p* < 0.001) compared to baseline. These results suggest that hybrid-type neuromuscular training may improve aspects of cardiometabolic health and antioxidant status in inactive overweight and obese women providing a time-efficient (~100 min/week) exercise approach in a real-world gym setting.

## 1. Introduction

The epidemic of overweight and obesity represents an excessive caloric intake over expenditure that leads to increased adiposity that causes health impairments. This condition now accounts for about one third of the world’s population, with women being alarmingly affected, since it is estimated that 21% of women will be obese by 2025 [1]. Obesity augments markedly the risk of developing cardiovascular disease (CVD), insulin resistance, type 2 diabetes mellitus, hypertension, respiratory diseases, and cancer that are not only the leading causes of death presently [2] but they also represent a substantial burden for the health care systems worldwide reaching, only in the United States, USD 149.4 billion annually [3].

In obesity, adipocytes expand in their number and size due to a progressive accumulation of triglycerides causing a rise of immune cell infiltration into adipose tissue, fibrosis and promotion of a pro-inflammatory state [4] which stimulates the generation of reactive oxygen species (ROS) [5]. Increased ROS levels may cause insulin resistance by triggering redox-sensitive pro-inflammatory cascades such as nuclear factor kappa B (NF-κB) and by altering the phosphorylation of the insulin receptor substrate 1 (IRS-1) [6]. These events promote IRS-1 degradation, faulty insulin signaling and impaired insulin-stimulated glucose uptake via protein modification and by triggering the mitogen activated protein kinase (MAPK) signaling [6].

Obesity may be largely prevented and managed through healthy nutrition and regular physical activity and exercise. Although physical inactivity is a major risk factor for developing obesity and related noncommunicable diseases [7] almost one third of the adult population [8] and approximately half of Caucasian women in developed countries [9] are classified as physically inactive. Currently, >300 min/week of moderate-intensity continuous cardiovascular training (MICT) and 2–3 bouts of moderate-intensity resistance training (RT) (>13 MET–hours/week) are required for a clinically meaningful weight loss aiming to improve several cardiometabolic health markers [10,11]. The addition of RT to weight loss programs further improves performance, lean body mass, resting metabolic rate and caloric expenditure and may reduce cardiometabolic risk in the overweight/obese [12]. Although these exercise modes induce weight loss, improve cardiometabolic risk and IR and reduce systemic oxidative stress [13,14], they are characterized by low long-term adherence because they are time-consuming and less motivating [15]. Contrarily, high-intensity interval training (HIIT) has been reported as a popular exercise mode [16] introducing cardiovascular activities that are more time-efficient, less monotonous and more enjoyable [17]. Additionally, such a training modality has been associated with increased adherence rates [17], substantial weight loss, and improved cardiorespiratory capacity, body composition, resting metabolic rate (RMR), and muscle mitochondrial metabolism even in the absence of a diet intervention in adults with obesity [18,19,20,21,22]. HIIT appears to improve arterial blood pressure, lipid profile, and glycemic regulation similarly to, if not superior, MICT despite the reduced time commitment [17,22].

Recent studies, however, revealed that HIIT-like protocols incorporating multiplanar, neuromuscular exercises performed in a circuit fashion, also called hybrid training protocols, may induce not only weight and fat loss, but they can also improve body composition, cardiorespiratory (CRF) and musculoskeletal fitness as well as several psychological health indicators related to exercise using only ~100 min/week [23,24,25]. Hybrid-type training is a new multicomponent exercise mode activating simultaneously both the cardiovascular and the musculoskeletal system throughout the same exercise session, at various intensities, using both muscle-strengthening exercises and dynamic cardiovascular activities [23]. Here, we hypothesized that this type of protocol may also improve cardiometabolic risk and reduce systemic oxidative stress in women with overweight and obesity. Therefore, this investigation aimed to examine whether a 5-month neuromuscular, circuit-type HIIT protocol could affect (i) resting cardiovascular function, (ii) blood lipid profile, (iii) glycemic control, (iv) cardiometabolic risk and (v) redox status of inactive women with overweight and obesity.

## 2. Materials and Methods

### 2.1. Participants and Experimental Design

**Figure 1:**
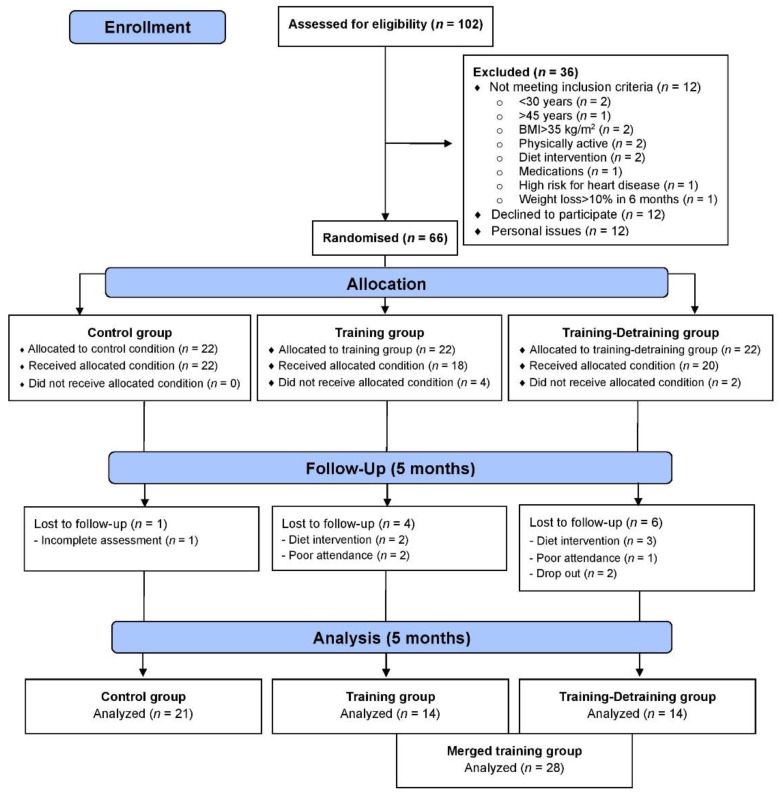
CONSORT flow diagram of the study.

This study is a part of a larger 10-month training-detraining randomized controlled trial (DoIT) based on a 3-group, repeated measures design in accordance with the Consolidated Standards of Reporting Trial (CONSORT) guidelines (Figure 1). DoIT trial was registered at clinicaltrials.gov as NCT03134781 and its purpose and methodology are reported elsewhere [25]. In this work, data upon secondary variables (cardiometabolic health, oxidative stress, and redox status) are presented. A preliminary power analysis (effect size >0.55, probability error of 0.05, 2-tailed alpha level, power of 0.9) using the G*Power 3.0.10 program revealed that a sample of 36–40 participants was necessary to identify statistically significant trial effects. Initially, 102 apparently healthy premenopausal women (30–45 years) with overweight and obesity were approached using fliers posted in the local community, social media and by word of mouth, and 66 were recruited. Randomization was attained by a random-numbers table (allocation sequence conducted by an independent researcher and was concealed until interventions were assigned).

**Figure 2:**
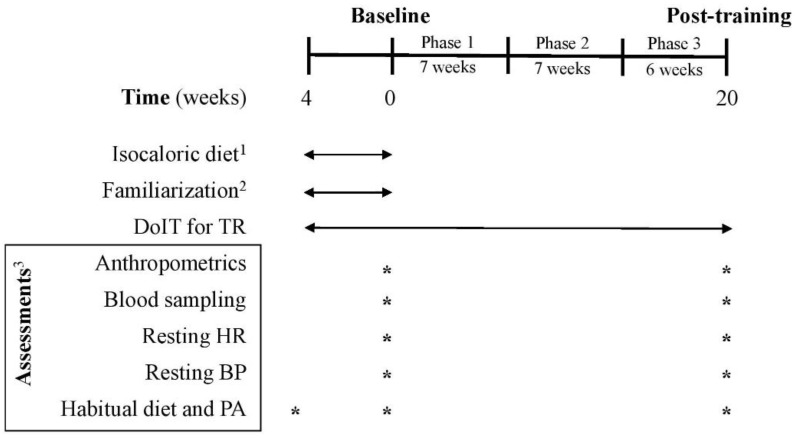
Experimental flowchart. BP: blood pressure; C: control group; DoIT: exercise training program; HR: heart rate; PA: physical activity; TR: training group. ^1^ for C and TR (4-week adaptive period); ^2^ only for TR; ^3^ for C and TR.

Participants in the merged TR trained for 5 months, whereas volunteers in C participated only in measurements. Assessments and blood sampling (Figure 2) were performed at pre- and post-training (5 days after the last training session). Participants were non-smokers, inactive (no structured exercise for ≥6 months prior to the study), not on medication, diet, or nutritional supplementation, medically cleared for participation in an exercise intervention and provided a written informed consent. Data were used only from participants with an attendance rate ≥80% and did not modify their dietary intake and PA levels during the intervention. The Institutional Review Board of the Department of Physical Education and Sport Sciences of the University of Thessaly (protocol ID: 1025/15-7-2015) approved the methods, procedures, and ethics of this study. Procedures agreed with the 1975 Declaration of Helsinki as revised in 2013. This study was registered on clinicaltrials.gov as NCT03134781.

### 2.2. Exercise Training Program

Three supervised weekly training sessions (of 5–10 participants/session) were performed on nonconsecutive days in a real-world gym setting. DoIT training included a circuit-type program using progressively loaded fundamental movement patterns and integrating neuromotor exercise training into an interval fashion as described [25]. A movement-based programming adapted for inactive persons with overweight and obesity was implemented and all exercises were complex movements executed in all planes of motion using nontraditional training implements (Table S1) [26]. Briefly, the training regimen was a hybrid of both aerobic- and resistance-based exercises of low volume (<30 min/session; 3 days/week) and incorporated low- to moderate-impact cardiovascular drills and compound RT exercises into an intermittent manner. Such an intermittent-based, multicomponent exercise mode in which both the cardiovascular and musculoskeletal systems are engaged simultaneously, at various intensities, throughout a single session cannot be classified as either MICT, or HIIT or RT [23,24,25]. Participants were encouraged to perform as many repetitions as possible for each exercise aiming to maintain the exercise intensity at levels higher than 75% of maximal heart rate (MHR). Heart rate (HR) and rate of perceive exertion (RPE) were monitored and recorded with telemetry (Polar Team Solution, Polar Electro-Oy, Kempele, Finland) and the Borg scale (6–20), respectively. After a 4-week adaptive and familiarization period, training had 3 phases of progressive intensity; i.e., phase 1 (weeks 1–7), phase 2 (weeks 8–14) and phase 3 (weeks 15–20) (Figure 2). This program was of ~100 min mean weekly exercise volume with a net exercise time of 6.5–24.0 min/session (23–41 min total duration/session) and its intensity was maintained at 73–87% of MHR with an RPE of 14–16. Mean blood lactate concentration and average energy expenditure ranged from 8–12 mM, and 165–411 kcal, respectively as previously published (Table S2) [25].

### 2.3. Descriptives

Habitual PA and caloric intake were assessed using procedures previously reported using 7-day accelerometry (GT3X+, ActiGraph, Pensacola, FL, USA) and 7-day diet recalls, respectively [25]. Diet recalls were analyzed by a dietitian for energy and macronutrient intake using a nutrition analysis software (Science Fit Diet 200A, Science Technologies, Athens, Greece). PA and caloric intake assessments were performed at baseline and after 5 months of training (Figure 2).

### 2.4. Abdominal Obesity Indicators and Resting Cardiovascular Function

Waist and hip circumferences were measured using a Gullick II and the waist-to-hip ratio (WHR) was calculated as reported [27,28]. Volunteers were asked to abstain from any vigorous physical activity and to not consume alcohol or caffeine products for 24 h before reporting to the laboratory in the morning (07:00–09:00 a.m.) after an overnight fast. A physician measured resting heart rate (RHR) by pulse palpation for 60 sec as well as blood pressure using a kit of arm sphygmomanometer and a stethoscope (Precisa N R-1362, Riester, Jungingen, Germany) as described [10]. Blood pressure was measured in a seated position in both arms twice with 1 min break between measurements. No average arm-to-arm differences were observed, and the median of all measurements was reported as the value in blood pressure. Mean arterial pressure (MAP) was calculated using the following equation: MAP = [SBP + (2 × DBP)]/3 [29].

### 2.5. Blood Sampling and Assays

Blood samples were drawn from an antecubital arm vein with participants seated at the same time of day (07:00–08:00 a.m.) to prevent circadian rhythm variations. For plasma separation, a blood portion was collected in tubes with EDTA and centrifuged (1370× *g*, 10 min, 4 °C), the supernatant was transferred into Eppendorf tubes and stored at −80 °C for later measurement of protein carbonyls (PC) and total antioxidant capacity (TAC). Packed erythrocytes were diluted with distilled water (1:1 *v*/*v*), mixed vigorously, and centrifuged (4000× *g*, 15 min, 4 °C) for red blood cell lysate (RBCL) preparation and the resultant supernatant was transferred into Eppendorf tubes and stored at −80 °C for later analysis of reduced glutathione (GSH) and catalase activity (CAT). Another blood portion was collected in serum tubes containing coagulant factor, left at room temperature for 20 min to clot, centrifuged (1370× *g*, 10 min, 4 °C) for serum separation with the supernatant transferred into Eppendorf tubes and stored at −80 °C for later determination of serum triglycerides (TG), total cholesterol (TC), and high-density lipoprotein cholesterol (HDL). PC, TAC, GSH and CAT were measured with spectrophotometric assays on a Hitachi 2001 UV/VIS (Hitachi Instruments Inc., San Jose, CA, USA) as previously described [30]. Hemoglobin in RBCL was measured on the same spectrophotometer with a commercially available kit (#60230, Dutch Diagnostics BV, Zutphen, The Netherlands). Commercially available kits (Human) were used for measuring TG (#10724), TC (#10028), and HDL (#10018) (P. Zafiropoulos, Athens, Greece) on an automated clinical chemistry analyzer Z1145 (P. Zafiropoulos Diagnostica, Athens, Greece). Fasting glucose (FG) and insulin (FI) levels in serum were determined using a commercially available insulin (Human) ELISA kit (#KA4088 and #KA3810, respectively, Abnova Corporation, Taipei, Taiwan) as previously reported [31]. Low-density lipoprotein cholesterol (LDL) concentration was calculated according to the equation LDL = TC − HDL − (TG/5) [32]. Atherogenic index (AI) and homeostatic model assessment of insulin resistance (HOMA-IR) were calculated as previously published [33]. All assays were performed in duplicates on the same day. The inter- and intra-assay coefficients of variability for all assays ranged from 3.4% to 6.9% and from 3.8% to 7.3%, respectively.

### 2.6. Risk Scores

A continuous risk score assessment scale (MetS z-score) was used to identify changes in MetS risk factors following training. The MetS z-score was previously validated to evaluate cardiometabolic risk in middle-aged women using HDL, TG, FG, waist circumference (WC) and MAP values as previously published [34]. The 10-year and 30-year cardiovascular disease (CVD) risks were estimated from the Framingham risk scores using the risk factors of age, sex, TC, HDL, smoking history, BP, and diabetes mellitus [35,36].

### 2.7. Statistical Analyses

Data normality was verified using the Shapiro–Wilk test. A 2 × 2 (condition × time) mixed ANOVA with a Bonferroni post-hoc test was used to analyze the data. Statistical significance was set at *p* < 0.05. Effect sizes (ES) and confidence intervals (CI) were calculated for all dependent variables using the Hedge’s g method corrected for bias. ES was interpreted as none, small, medium-sized, and large for values 0.00–0.19, 0.20–0.49, 0.50–0.79 and ≥0.8, respectively. Because of the variability of the change score in TR was greater than that in C, training responsiveness was analyzed using the number of differential responders relative to the ratio of variance in TR and C groups providing multiple differential responder groups (adverse, low, average, and high) as previously reported [37]. Data were analyzed using the SPSS 23.0 software (IBM Corp., Armonk, NY, USA). Results are presented as mean ± standard deviation (SD) and percentage change (Δ%).

## 3. Results

**Figure 3:**
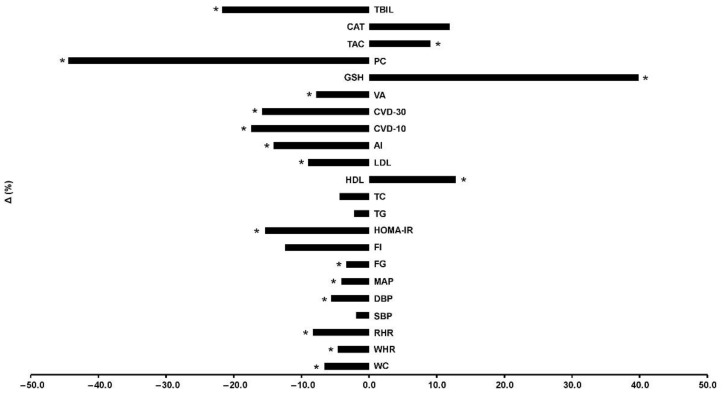
Percentage changes [Δ% = (Post − Pre)/Pre*100] in cardiometabolic health, redox status, and oxidative stress indicators in TR following a 5-month intervention. AI: atherogenic index; CAT: catalase; CVD-10: 10-year cardiovascular disease risk; CVD-30: full 30-year cardiovascular disease risk; DBP: diastolic blood pressure; FG: fasting glucose; FI: fasting insulin; GSH: glutathione; HDL: high-density lipoprotein; HOMA-IR: homeostatic model assessment of insulin resistance; LDL: low-density lipoprotein; MAP: mean arterial pressure; PC: protein carbonyls; RHR: resting heart rate; SBP: systolic blood pressure; TAC: total antioxidant capacity; TBIL: total bilirubin; TC: total cholesterol; TG: triglycerides; VA: vascular age; WC: waist circumference; WHR: waist-to-hip ratio. * Significant differences with baseline levels (*p* < 0.05).

**Figure 4:**
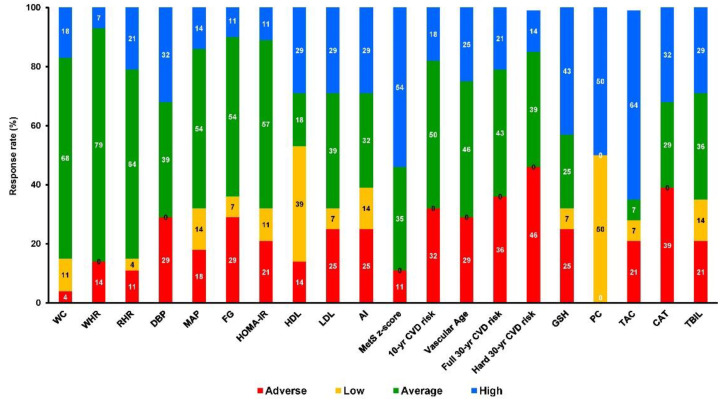
Multiple differential responder groups to exercise in TR following a 5-month intervention. AI: atherogenic index; CAT: catalase; CVD-10: 10-year cardiovascular disease risk; CVD-30: full 30-year cardiovascular disease risk; DBP: diastolic blood pressure; FG: fasting glucose; FI: fasting insulin; GSH: glutathione; HDL: high-density lipoprotein; HOMA-IR: homeostatic model assessment of insulin resistance; LDL: low-density lipoprotein; MAP: mean arterial pressure; PC: protein carbonyls; RHR: resting heart rate; SBP: systolic blood pressure; TAC: total antioxidant capacity; TBIL: total bilirubin; TC: total cholesterol; TG: triglycerides; VA: vascular age; WC: waist circumference; WHR: waist-to-hip ratio.

Participants were allocated to 3 groups and finally 49 completed the trial (Figure 1): control (C, *n* = 21), training (TR, *n* = 14, trained for 10 months), and a training–detraining group (TRD, *n* = 14, who trained for 5 months and then entered a detraining period for another 5 months). In this study, we present the results on secondary outcome variables; i.e., cardiometabolic health and redox status (our primary outcomes were body mass, body composition, resting metabolic rate and performance variables following 10 months of intervention) after 5 months of training using 2 arms (data from the training and detraining group were pooled together since they followed exactly the same training program—no detraining data are presented here due to failure to obtain blood samples at 10 months of the intervention) (Figure 1). Participants’ characteristics are shown in Table 1. Percentage changes (Δ%) in all parameters in TR are presented in Figure 3. Multiple differential responder groups to exercise in TR are illustrated in Figure 4.

No differences existed between groups in all variables at baseline. An 88% adherence and a 7% dropout (*n* = 2) rates were recorded for TR. No injuries or other adverse effects were noted. Absolute changes in all variables in both groups are shown in Table 2 and Δ% in TR is shown in Figure 3. Results are described in brackets as Δ%, 95% CI, ES, and *p* level. No changes were noted in C for all variables.

### 3.1. Abdominal Obesity Indicators and Resting Cardiovascular Function

TR showed lower WC (−6.3%, 1.954–10.224, −0.83, *p* = 0.005) and WHR (−4.2%, 0.007–0.065, −0.75, *p* = 0.015) compared to C at post-training. TR reduced WC (−6.6%, 5.414–7.515, −0.74, *p* < 0.001) and WHR (−4.6%, 0.027–0.051, −0.71, *p* < 0.001) compared to baseline values. The overall response rate for WC and WHR was 96% and 86%, respectively. TR resulted in greater changes of the RHR (−7.0%, 0.187–11.146, −0.55, *p* = 0.043) and MAP (−4.5%, 0.416–7.651, −0.65, *p* = 0.03) while demonstrating a trend for reduction in DBP (−5.4%, 0.048–8.334, −0.56, *p* = 0.053) compared to C. TR reduced RHR (−8.3%, 5.085–8.487, −0.72, *p* < 0.001), DBP (−5.6%, 2.722–5.921, −0.46, *p* < 0.001) and MAP (−4.1%, 2.536–4.743, −0.46, *p* < 0.001) but not SBP despite a reduction (−1.9%) from baseline values. The overall response rate for RHR, DBP, and MAP was 89%, 71%, and 82%, respectively.

### 3.2. Glucose and Lipid Metabolism

TR reduced FG (−3.4%, 1.118–4.875, −0.33, *p* = 0.002) and HOMA-IR (−15.7%, 0.164–0.448, −0,41, *p* < 0.001). No statistically significant change was noted for FI even though the difference (−12.4%) between post-training and baseline levels. The overall response rate for glucose and HOMA-IR was 71% and 79%, respectively. TR increased HDL (+18.2%, 0.611–11.043, 0.63, *p* = 0.029) and reduced AI (−17.0%, 0.024–2.044, −0.58, *p* = 0.045) relative to C. TR increased HDL (+12.8%, 2.139–6.383, 0.49, *p* < 0.001), reduced LDL (−8.6%, 2.641–21.545, 0.33, *p* = 0.013) and AI (−14.1%, 0.444–1.213, −0.48, *p* < 0.001) compared to baseline levels. The reductions in TG (−2.2%) and TC (−4.4%) were not statistically significant. The overall response rate for HDL, LDL, and AI was 86%, 75%, and 75%, respectively.

### 3.3. Antioxidant Capacity and Oxidative Stress

TR demonstrated greater changes in PC (−45.7%, 1.742–6.510, −0.99, *p* = 0.001), CAT (+15.2%, 4.726–61.436, 0.67, *p* = 0.023), and TAC (+11.4%, 0.033–0.144, 0.92, *p* = 0.002) compared to C at post-training. TR reduced PC (−44.5%, 0.248–0.540, −0.84, *p* < 0.001) and TBIL (−21.6%, 0.050–0.149, −0.71, *p* < 0.001) and increased GSH (+39.8%, 0.058–0.156, 0.47, *p* < 0.001) and TAC (+9.1%, 0.043–0.100, 0.76, *p* < 0.001) compared to baseline values. Although no time-dependent differences were detected in both groups, CAT values were greater in TR (+15.2%, 4.726–61.436, 0.67, *p* = 0.023) compared to post-training. The overall response rate for PC, GSH, TAC, CAT, and TBIL was 100%, 75%, 79%, 61%, 79%, respectively.

### 3.4. Risk Scores

TR showed favorable changes in the MetS z-score (−222%, 2.840–9.215, −1.08, *p* < 0.001) relative to C at post-training. TR reduced the MetS z-score (−123%, 0.513–1.794, −0.52, *p* = 0.001), the 10-year CVD risk (−17.4%, 0.093–0.671, −0.35, *p* = 0.011), the full 30-year CVD risk (−15.8%, 0.885–3.615, −0.36, *p* = 0.002), the hard 30-year CVD risk (−17.6%, 0.283–2.003, −0.32, *p* = 0.01) and the VA (−7.9%, 1.140–4.717, −0.37, *p* = 0.002) compared to baseline levels. The overall response rate for MetS z-score, 10-year CVD risk, full 30-year CVD risk, hard 30-year CVD risk, and VA was 89%, 68%, 64%, 54%, and 71%, respectively.

## 4. Discussion

A 5-month hybrid-type interval training protocol conducted under real-world conditions resulted in a marked reduction of cardiometabolic risk factors and improved redox status in previously inactive women with overweight and obesity. The present study focuses on a cohort characterized by a higher cardiometabolic risk [2], elevated oxidative stress levels [38], and lower antioxidant capacity [39]. Considering that data from long-term (>12 weeks) nontraditional HIIT interventions are currently critically limited [17,40], this study reveals that a protocol of limited net exercise time and overall weekly volume is effective for improving various cardiometabolic health indices in the fastest growing population worldwide [1].

The inclusion of muscle-strengthening activities to such intermittent-based exercise programs appears to increase muscular strength (+14%), fat-free mass (+1.3%), resting metabolic rate (+7.4%) and energy expenditure (900 kcal/week) resulting in improved body mass (−4.2%), body fat (−6.9%), and BMI (−4.5%) as we previously published elsewhere [25]. Such physiological adaptations have been reported significantly beneficial for improving cardiometabolic health in previously inactive overweight and obese women [41,42]. In addition, the HIIT-type approach used in our protocol may be beneficial for increasing cardiorespiratory fitness (+18.5%) as we reported elsewhere [25]. Such increases in maximal aerobic capacity may be occurred because of favorable mitochondrial adaptations regularly observed for other HIIT protocols in various populations, including the obese [17,21]. Interestingly, these findings are observed even though most variables demonstrated normal baseline levels since all participants were apparently healthy, premenopausal overweight, and obese women. However, all participants were central obese individuals according to the WC and WHR baseline levels, which may be a critical point associated with impaired cardiometabolic health and increased risk of developing MetS [43]. Although the present study did not examine the effects of DoIT on irisin and brown adipose tissue that is well known to be able to modulate the cardiovascular risk, the positive alterations in several markers reported here may be potentially associated with the beneficial role of HIIT-like interventions in cardiometabolic health enhancement [44,45]. This observation may indicate the preventive role of such a hybrid-type exercise mode in any future cardiometabolic health complications commonly occurred in postmenopausal overweight and obese women [46].

### 4.1. Resting Cardiovascular Function Responses

Although RHR is not considered an independent CVD risk factor, the evidence concerning negative impact of raised RHR on CVD mortality is constantly increasing [47]. The present findings show that DoIT induced a mean reduction of 7.7% (−6.2 bpm), which is similar to the reduction induced by MICT, HIIT, RT and combined MICT and RT protocols (−4.5% to −6.8% or −2.2 to −4.3 bpm) [47]. Although this study did not examine the mechanisms of this RHR reduction, it is well known that regular exercise decreases the intrinsic heart rate and the response to beta-adrenergic stimulation while increasing the resting parasympathetic tone [47].

Blood pressure is a major risk factor for developing CVD with MAP being an independent predictor of stroke due to a rise in systemic vascular resistance and resting cardiac output [48]. Exercise training has been widely reported as an effective strategy to manage blood pressure in various populations (10). However, the optimal exercise characteristics for blood pressure management in individuals with overweight and obesity are still unclear. DoIT reduced DBP (−5.6% or −5.4 mmHg) and MAP (−4.3% or −3.9 mmHg) in inactive normotensive women with obesity, which coincides with reductions observed following MICT in hypertensive (4–6%), prehypertensive (2–4%), and normotensive (1–2%) individuals [10]. HIIT has been associated with a greater (−5% vs. −3%) decline of blood pressure than MICT in adults with overweight and obesity [48] and this fact corroborates further our findings for HIIT-type protocols. Compared with DoIT, the HIIT protocol in that study used a similar work-to-rest ratio (1:1), frequency (3x/week), intensity (~90% MHR, RPE~15) and duration (~30 min), but a different modality (cycling) and recovery type (active). Moreover, DoIT triggered greater changes in blood pressure compared with RT (SBP/DBP: 0.0/−0.9 mmHg) and combined training (SBP/DBP: +0.9/−1.5 mmHg) in normotensive adults. In fact, DoIT induced adaptations similar to those observed in studies with hypertensive and prehypertensive populations using MICT, RT, or combined training [49]. Improved endothelial function and autonomic regulation as well as reduced oxidative stress are considered to be the potential physiological mechanisms responsible for these adaptations in blood pressure following HIIT-type programs [48]. These results support the rationale of considering DoIT-like protocols as exercise approaches for blood pressure management in adults with overweight and obesity.

### 4.2. Glucose Metabolism Responses

Insulin resistance has been documented as one of the leading causes of cardiovascular complications in those with impaired metabolic health that is associated with increased oxidative stress and inflammation [5]. DoIT elicited a marked reduction in FG (−3.4%) and HOMA-IR (−15.7%) over a 5-month intervention. HIIT has been shown to reduce HOMA-IR by approximately 20% in those with impaired glycemic control [50] and appears to be more effective than MICT in improving glucose metabolism [40]. Moreover, RT and combined training have been documented as exercise strategies capable of inducing a positive impact on glycemic control and other metabolic health risk factors associated with CVD [47]. Combined training seems to be superior to RT alone, but HIIT has been reported as the most effective modality for reducing insulin resistance in sedentary adults with obesity [50]. As such, DoIT was designed to include passive rest periods (20–40 s) enabling participants to accumulate time at exercise intensities challenging the cardiovascular system more than other exercise modalities in sedentary adults with obesity [51,52]. DoIT has been shown to markedly improve CRF (+27%) while inducing increased lactate concentration levels (9–12 mM) [25]. Lactate has been implicated in HIIT-induced metabolic adaptations via regulation of molecular mechanisms responsible for regulating whole-body glucose homeostasis such as the raised facilitated glucose transporter member 4, increased aerobic enzyme capacity and mitochondrial biogenesis associated with improved maximal aerobic capacity [50]. Additionally, populations with overweight and obesity are likely to exhibit poor glycemic control and impaired blood lipid profile associated with declined CRF and raised ROS levels due to a chronic inflammation of adipose tissue by stimulating the immune system [5]. Thus, DoIT-like exercise protocols that incorporate HIIT and RT into a time-efficient regimen may be an effective training alternative for improving metabolic health and antioxidant capacity in persons with obesity because of potential intracellular metabolic changes that have been documented as essentials elements for preventing obesity progression [50]. Furthermore, abdominal obesity has been linked to the development of dyslipidemia, hypertension, type 2 diabetes, and CVD due to the visceral fat accumulation that negatively affects ROS levels and causes insulin resistance IR by activating NF-κB resulting in IRS-1 malfunction and degradation and impaired insulin-stimulated glucose uptake [6]. In this study, central adiposity markers such as WC and WHR declined by −6.7% and −4.5%, respectively suggesting that this protocol may positively affect fat redistribution in adults with obesity [5].

### 4.3. Lipid Metabolism Responses

Cholesterol-lowering exercise training is critical for individuals with obesity in order to avoid future problems with dyslipidemia, a major modifiable cause of CVD and a very common health condition among populations with unhealthy weight and low CRF levels [10]. Changes in blood lipid profile are often small in apparently healthy adults with overweight or obesity. However, the cohort examined in the present study showed abnormal HDL (<40 mg/dL) and LDL (<130 mg/dL) values while TC values were in the higher levels within the normal range (~190 mg/dL) at baseline [53]. Considering that it has been well documented that CVD develops progressively in adults with obesity, it seems that prevention and not only treatment should be a high priority for this population [54]. In this investigation, 15 min of net exercise time and a total of l00 min weekly time commitment produced significantly changes in HDL (+18.1% or +5.8 mg/dL) and the AI (−17%) compared with controls while reduced LDL (−8.6% or −13.5 mg/dL) during a 5-month intervention in the absence of dietary restrictions. Specifically, 250–300 min/week of MICT consistently reduces LDL by 3–6 mg/dL without altering the HDL and TG blood levels [10]. RT appears to reduce LDL and TG concentrations by 6–9 mg/dL but with less consistency compared with MICT (10). Specifically, long-term (12–20 weeks) RT improved TC (−10%), LDL (−5% to −18%), HDL (+13%), TG (−28%), and AI (−8% to −22%) without diet in sedentary individuals [51,55]. According to current exercise prescription guidelines, combined training is highly recommended for improving the blood lipid profile in individuals with overweight and/or dyslipidemia [10]. However, the aforementioned exercise training approach seems a very time-consuming exercise strategy since it exceeds 300–400 min/week. In contrast, both short- (<12 weeks) and long-term (>12 weeks) HIIT protocols show no consistent evidence for their effects on lipid profile in populations with overweight and obesity [17]. HIIT and MICT demonstrate similar efficacy on lipid metabolism in this cohort [21,40]. These outcomes agree with our findings supporting the evidence from previous research underlying the inclusion of HIIT in the exercise prescription for adults with impaired blood lipid metabolism [52,56]. These changes may indicate greater cardiovascular and hemodynamic adaptations through autonomic nervous system adjustments associated with the positive alterations that we found in RHR, DBP, and MAP [57]. However, HIIT decreases blood flow in adipose tissue by up-regulating α2-adrenergic receptors at high catecholamine concentrations resulting in reduced fatty acid release into the circulation [17,40]. Nevertheless, the training method in our study resulted in positive changes in blood lipid profile and due to time-efficient approach it should be considered to be an effective method to produce health benefits in this cohort that demonstrated an unhealthy lipid profile at baseline.

### 4.4. Antioxidant Capacity Responses

Elevated oxidative stress and impaired redox status are frequently associated with obesity-induced inflammation playing a key role in enhancing several CVD risk factors [38]. DoIT elicited significant alterations in PC (−45%), GSH (+40%), TAC (+10%), CAT (+15%), and TBIL (−22%) therefore providing evidence that this type of protocols may be an effective tool for improving this physiological dysfunction [38]. Similar findings were observed following a 4-week HIIT protocol (2x/week, 85–90% MHR, 31.5 min, active recovery intervals) on oxidative stress and antioxidant capacity markers in postmenopausal sedentary women with obesity [58]. Furthermore, a 3-week HIIT cycling protocol totaling 22 min of pure high-intensity exercise time using 30-s bouts and 4-min active or passive recovery in active young men exhibited a reduction of 13% in PC and 26% in both TAC and CAT [13]. In contrast, HIIT and MICT failed to induce positive alterations in PC and TAC following a 12-week supervised intervention in women with type 2 diabetes [59]. Results in the present study could be explained by an up-regulation of the antioxidant enzymatic system as demonstrated by the rise in CAT. In fact, CAT and glutathione peroxidase activities have been associated with reduced ROS production in skeletal muscle in response to HIIT [52]. In addition, the inclusion of moderate to high-intensity RT exercises in DoIT may not affect the serum concentrations of cell adhesion molecules linked to impaired immune system [60]. Interestingly, there are very limited data reporting the effects of different exercise modes on serum TBIL levels in inactive populations with obesity. TBIL increases in response to strenuous exercise resulting in erythrocyte damage therefore promoting inflammation and ROS production [13]. However, in this study, TBIL decreased suggesting that such a type of low-volume protocols is safe despite its high intensity.

### 4.5. MetS and CVD Risk Responses

DoIT resulted in an impressive reduction in the MetS z-score (−123%) with an 89% response rate. Similar changes are reported for the 10-year (−17%), full 30-year (−16%), and hard 30-year (−18%) CVD risk scores as well as in the estimated VA (−8%). It is worth mentioning that the full and hard 30-year CVD risk scores presented baseline values within the risk category and normalized post-intervention [36]. The potential mechanisms responsible for these findings have not been elucidated, but it has been widely documented that they are mostly dependent on the training configurations of each regimen [52]. However, there is some debate regarding the value of cholesterol as a predictor of CVD and therefore an examination of the inflammatory pathway would also be necessary to categorically confirm CVD risk and modification with exercise. The exercise-induced cardiometabolic health adaptations to HIIT are comparable with those induced by MICT in individuals with obesity but the former is characterized by far less time commitment [56]. The substantial cardiovascular adaptations seen here and the neuromuscular increases that have previously been reported for this population [25] in response to DoIT have been associated with a risk reduction for type 2 diabetes, MetS, and CVD [50]. This inverse relationship may be due to the significant positive association of the above adaptations with insulin sensitivity contributing to the reduction of the MetS severity.

### 4.6. Limitations

Our study recruited sedentary Caucasian women with overweight and obesity and thus it is not possible to generalize the findings to other populations. Given that hybrid-type exercise approaches incorporate some of the most attractive modalities into real-world gym settings [61,62] further research is needed in several areas investigating such exercise protocols in males and other age and race groups for more prolonged periods (>6 months) as previously described [56,63]. Considering that GSH is considered to be one of the most important scavengers of ROS, and its ratio with oxidized glutathione (GSSG) may be used as a marker of oxidative stress, the present study did not address and quantify redox status ideally. In addition, PC as the only oxidative stress marker does not seem strong enough evidence to support in-depth such an assessment procedure.

## 5. Conclusions

The reported varied effects of DoIT on cardiometabolic health risk factors and redox status markers presented here may be critical for this population. DoIT promotes favorable changes in RHR and blood pressure, glycemic control, redox status, and MetS severity using a 3- to 4-fold lower weekly exercise volume and time commitment compared with traditional MICT. Moreover, a high-intensity interval neuromuscular training program seems to be a time-efficient and effective exercise training strategy for lowering CVD risk in previously inactive women with overweight and obesity. These results have public health implications as they suggest a hybrid-type, injury-free training modality integrating HIIT and functional RT into a small group format as a considerable novel approach that may be included in the future recommendations for prescribing exercise to individuals with overweight and obesity.

## Figures and Tables

**Table 1:** Participants’ characteristics.

	Pre			Post		
Variables	C	TR	*p*	C	TR	*p*
Age (yr)	36.0 ± 4.2	36.6 ± 4.6	0.644	36.0 ± 4.2	36.6 ± 4.6	0.644
Body mass (kg)	80.2 ± 8.9	78.1 ± 8.8	0.425	80.4 ± 7.7	74.8 ± 9.1	0.030
Body height (m)	1.65 ± 0.5	1.65 ± 0.5	0.748	1.65 ± 0.5	1.65 ± 0.5	0.748
BMI (kg/m^2^))	29.6 ± 3.0	28.7 ± 2.9	0.278	29.7 ± 2.7	27.5 ± 3.2	0.012
PA (steps/day)	6400 ± 1851	6600 ± 1537	0.695	6370 ± 1827	6649 ± 1712	0.587

BMI: body mass index C: control group; PA: physical activity; TR: training group. Values are expressed as mean ± SD.

**Table 2:** Mean changes in cardiometabolic health, redox status, and oxidative stress indicators during 5 months of training.

	C		TR	
Variables	Pre	Post	Pre	Post
WC (cm)	95.9 ± 5.3	96.1 ± 4.8	96.5 ± 8.7	90.1 ± 8.4 *^,†^
WHR	0.87 ± 0.04	0.87 ± 0.04	0.87 ± 0.05	0.83 ± 0.06 *^,†^
FG (mg/dL)	87.29 ± 10.15	87.56 ± 10.51	87.68 ± 10.02	84.69 ± 7.53 *
FI (mU/L)	8.89 ± 4.01	9.01 ± 3.52	8.97 ± 3.33	7.86 ± 3.04
HOMA-IR	1.93 ± 1.01	1.96 ± 0.91	1.95 ± 0.79	1.65 ± 0.66 *
TG (mg/dL)	93.5 ± 17.1	93.9 ± 17.0	91.3 ± 27.9	89.4 ± 27.1
TC (mg/dL)	179.4 ± 37.5	184.1 ± 38.9	187.1 ± 35.0	178.9 ± 36.5
HDL (mg/dL)	32.4 ± 8.7	32.1 ± 8.6	33.6 ± 8.0	37.9 ± 9.3 *^,†^
LDL (mg/dL)	128.3 ± 35.8	133.2 ± 37.4	135.2 ± 34.2	123.1 ± 37.5 *
AI	5.87 ± 1.83	6.07 ± 1.88	5.87 ± 1.79	5.04 ± 1.62 *^,†^
TBIL (mg/dL)	0.409 ± 0.162	0.398 ± 0.147	0.461 ± 0.150	0.361 ± 0.126 *
RHR (bpm)	80.2 ± 12.6	80.5 ± 12.9	81.6 ± 9.6	74.9 ± 7.0 *^,†^
SBP (mmHg)	116.1 ± 6.7	116.5 ± 6.5	114.9 ± 6.8	112.7 ± 9.8
DBP (mmHg)	76.8 ± 6.9	77.0 ± 5.4	77.2 ± 10.0	72.9 ± 8.3 *
MAP (mmHg)	89.9 ± 4.8	90.2 ± 3.7	89.8 ± 8.2	86.1 ± 7.6 *^,†^
GSH (mmol/g Hb)	0.282 ± 0.235	0.271 ± 0.223	0.269 ± 0.192	0.376 ± 0.249 *
PC (nmol/mg protein)	0.899 ± 0.511	0.904 ± 0.510	0.885 ± 0.574	0.491 ± 0.317 *^,†^
CAT (U/mg Hb)	219.1 ± 61.1	217.1 ± 60.7	223.6 ± 54.1	250.2 ± 37.7 ^†^
TAC (mmol DPPH/L)	0.783 ± 0.095	0.774 ± 0.097	0.791 ± 0.092	0.863 ± 0.094 *^,†^
MetS z-score	−0.80 ± 1.98	−0.65 ± 1.79	−0.94 ± 1.98	−2.09 ± 2.37 *^,†^
10-year CVD risk (%)	2.1 ± 0.8	2.3 ± 0.9	2.3 ± 1.3	1.9 ± 0.9 *
Vascular age (yr)	36.5 ± 5.7	37.2 ± 6.3	37.3 ± 7.9	34.4 ± 7.4 *
Full 30-year CVD risk (%)	13.5 ± 4.7	14.2 ± 5.3	14.3 ± 6.9	12.0 ± 5.6 *
Hard 30-year CVD risk (%)	5.9 ± 2.3	6.3 ± 2.6	6.5 ± 3.9	5.4 ± 2.9 *

AI: atherogenic index; C: control group; CAT: catalase; CVD: cardiovascular disease; DBP: diastolic blood pressure; FG: fasting glucose; FI: fasting insulin; GSH: reduced glutathione; HDL: high-density lipoprotein; HOMA-IR: homeostatic model assessment of insulin resistance; LDL: low-density lipoprotein; MAP: mean arterial pressure; MetS: metabolic syndrome; PC: protein carbonyls; RHR: resting heart rate; SBP: systolic blood pressure; TAC: total antioxidant capacity; TBIL: total bilirubin; TC: total cholesterol; TG: triglycerides; TR: training group; WC: waist circumference; WHR: waist-to-hip ratio. * Significant differences with baseline levels (*p* < 0.05); ^†^ Significant differences with C (*p* < 0.05).

## Data Availability

All relevant data in the current study are available from the corresponding author on request.

## Supplementary Materials

The following are available online at www.mdpi.com/article/10.3390/antiox10101601/s1, Table S1: The exercises of the training protocol, Table S2: The characteristics of the training protocol.

## Abbreviations

BMI	body mass index
CAT	catalase
C	control group
CI	confidence intervals
CVD	cardiovascular disease
DBP	diastolic blood pressure
ES	effect sizes
FI	fasting insulin
FG	fasting glucose
GSH	reduced glutathione
HDL	high-density lipoprotein
HIIT	high-intensity interval training
HOMA-IR	homeostasis model assessment of insulin resistance
IRS-1	insulin receptor substrate 1
LDL	low-density lipoprotein
MAP	mean arterial pressure
MAP	mitogen activated protein kinase
MET	metabolic equivalent of task
MetS	metabolic syndrome
MICT	moderate-intensity continuous training
MHR	maximal heart rate
NF-κB	nuclear factor kappa B
PC	protein carbonyls
ROS	reactive oxygen species
RPE	rate of perceived exertion
RT	resistance training
SBP	systolic blood pressure
TAC	total antioxidant capacity
TBIL	total bilirubin
TC	total cholesterol
TG	triglycerides
TR	training group
WC	waist circumference
WHR	waist-to-hip ratio
