# Supplementary material for: Hybrid Neuromuscular Training Improves Cardiometabolic Health and Alters Redox Status in Inactive Overweight and Obese Women: A Randomized Controlled Trial
Source: Antioxidants (Basel). 2021 Oct 12;10(10):1601. doi: 10.3390/antiox10101601 (PMC8533161; doi:10.3390/antiox10101601)
Supplement: Supplementary file 1 [file antioxidants-10-01601-s001.zip › antioxidants-1366044-supplementary.pdf]

antioxidants

MDPI

Article

Hybrid Neuromuscular Training Improves Cardiometabolic Health and Alters Redox Status in Inactive Overweight and Obese Women: A Randomized Controlled Trial

Supplementary Materials

Table S1. The exercises of the training protocol.

Adjunct Modalities	Phase 1	Phase 2	Exercises Phase 3
1 – Balance Ball	Over dome ankle touch	Straddle jump	Split jack
2 – Suspension Exercise Device	Neutral grip row	Wide grip row	Y deltoid raise
3 – Kettlebell	Sumo deadlift	Sumo deadlift high pull	Two-arm swing
4 – Bodyweight	Straight-arm plank	Forearm plank	Straight-arm reverse plank
5 – Speed Ladder	Low knee skip	Lateral shuffle	Heel flick
6 – Battling Rope	Bilateral wave	Alternating wave	Side-to-side wave
7 – Medicine Ball	Alternating static lunge	Forward lunge with press	Lunge to chest pass
8 – Foam Roller	Forearm plank	Forearm plank with leg lift	Shifting Plank
9 – Bodyweight	Jumping jack	Split jack	Ice skater
10 – Resistance Band with Stick	Squat to overhead press	Lateral shuffle press	Hockey slap shot
11 – Resistance Band		Squat row	Reverse fly with lunge
12 – Medicine Ball			Squat throw

Table S2. The characteristics of the training protocol.

Training Parameters	Phase 1 (Weeks 1-7)	Phase 2 (Weeks 8-14)	Phase 3 (Weeks 15-20)
Session duration (min)	23.0	38.0	41.0
Effort time (min)[a]	6.66	16.5	24.0
Recovery time (min)[b]	16.34	21.5	17.0
Work-to-rest ratio	1:2	1:1	2:1
Work interval (sec)	20.0	30.0	40.0
Rest interval (sec)	40.0	30.0	20.0
Exercises amount	10	11	12
Rounds	2	3	3
Rest time/round (min)	3.0	2.5	2.5
Movement number[c]	As many as possible	As many as possible	As many as possible

[a] Effort time = session duration – recovery time.
[b] Recovery time = session duration – effort time.
[c] Maximal number of movements during efforts time.
